# Lumen-Apposing Metal Stents for Endoscopic Drainage of Intra-abdominal Abscesses: A Systematic Review

**DOI:** 10.7759/cureus.104256

**Published:** 2026-02-25

**Authors:** Iñaki Guasque Gil, Nicole Elbjorn Medina, Julianne Villa Trejo, César Villa Jirash

**Affiliations:** 1 Department of General Surgery, Hospital General de Especialidades "Dr. Javier Buenfil Osorio", Campeche, MEX; 2 Department of General Surgery, Instituto Nacional de Ciencias Médicas y Nutrición Salvador Zubirán, Ciudad de México, MEX; 3 Department of General Surgery, Universidad Saint Luke, Ciudad de México, MEX; 4 Department of General Surgery, Hospital San Ángel Inn, Ciudad de México, MEX

**Keywords:** abscess management, endoscopic drainage, endoscopic ultrasound, intra-abdominal abscess, lams, lumen-apposing metal stents, minimally invasive drainage

## Abstract

Intra-abdominal abscesses require timely source control, most commonly achieved through image-guided percutaneous drainage. However, percutaneous access may be unfeasible or ineffective in selected cases due to anatomical constraints, interposed organs, or prior drainage failure. Endoscopic ultrasound (EUS)-guided drainage using lumen-apposing metal stents (LAMS) has emerged as an alternative minimally invasive strategy, although evidence supporting its use in non-pancreatic intra-abdominal abscesses remains limited and heterogeneous.

This systematic review was conducted in accordance with Preferred Reporting Items for Systematic Reviews and Meta-Analyses (PRISMA) guidelines. A comprehensive search of PubMed/MEDLINE was performed from database inception through January 2024 to evaluate the feasibility, effectiveness, and safety of EUS-guided LAMS for non-pancreatic intra-abdominal abscesses. The primary objective was to translate these findings into a conservative, anatomy-driven approach to support clinical decision-making. A total of 16 studies involving 99 patients were identified, including observational studies, case series, and case reports; studies focusing exclusively on pancreatic fluid collections were excluded. Methodological quality and risk of bias were assessed using the Joanna Briggs Institute (JBI) checklists. Primary outcomes included technical and clinical success, while secondary outcomes comprised adverse events and reintervention rates.

Sixteen studies met the inclusion criteria, consisting predominantly of retrospective series and case reports. Reported technical success ranged from approximately 92.5% to 100%, with clinical success rates between 88% and 100%. Adverse events were infrequent and generally mild, occurring in fewer than 10% of cases; these were predominantly mild, including transient abdominal or rectal discomfort, minor bleeding, and occasional stent migration. Most abscesses were pelvic or postoperative collections, commonly managed via transrectal or transcolonic access routes. Evidence supporting transgastric or transduodenal drainage was limited to carefully selected upper abdominal collections.

EUS-guided drainage using LAMS appears to be a feasible and effective option in carefully selected patients with non-pancreatic intra-abdominal abscesses, particularly when percutaneous drainage is unfeasible or has failed. Success depends on strict apposition to the gastrointestinal lumen and adherence to the 'shortest-safe-path' principle, making it a viable rescue strategy after conventional drainage failure. Given the limitations of the available evidence, including small sample sizes and predominantly observational designs, this technique should be applied cautiously, with rigorous patient selection, strict anatomical considerations, and a predefined strategy for stent management and removal.

## Introduction and background

Intra-abdominal abscesses (IAAs) represent a common and potentially severe complication of gastrointestinal diseases and postoperative conditions, frequently necessitating prompt source control in addition to antimicrobial therapy. Image-guided percutaneous drainage (PD) is considered the first-line interventional approach when feasible due to its minimally invasive nature and favorable success rates [1,2].

However, PD may be limited or unsuccessful in certain scenarios, including deep pelvic abscesses, complex postoperative anatomy, interposed bowel loops, or the absence of a safe percutaneous access window. In these situations, failure of PD may necessitate repeated interventions or surgical drainage, thereby increasing patient morbidity and overall healthcare utilization [2,3].

Endoscopic ultrasound (EUS)-guided drainage has emerged as an alternative minimally invasive option for selected IAAs that are anatomically favorable and closely apposed to the gastrointestinal lumen. Real-time imaging and precise transluminal access enable effective drainage when conventional approaches are not feasible or have failed [4]. The introduction of lumen-apposing metal stents (LAMS), designed to create a stable, wide-diameter conduit between the gastrointestinal tract and the target collection, has further expanded the therapeutic role of EUS-guided drainage beyond pancreatic fluid collections [5-7].

Over the past decade, case reports, case series, and multicenter retrospective studies have described encouraging technical and clinical outcomes of EUS-guided drainage using LAMS for non-pancreatic IAAs, particularly pelvic, paracolic, and postoperative collections in carefully selected patients, particularly in cases where anatomical barriers or lack of a safe radiologic window make percutaneous access impossible [8-10].

Nevertheless, the available evidence remains heterogeneous and predominantly observational. Substantial variability exists in abscess etiology, anatomical location, access routes, and outcome definitions, and no standardized framework currently guides clinical decision-making in this setting.

Accordingly, a qualitative systematic review was conducted to synthesize the available evidence regarding EUS-guided LAMS drainage for non-pancreatic IAAs. In recognition of the limitations of the existing literature, a conservative, anatomy-driven practical approach is outlined to support clinical decision-making rather than to establish formal guideline recommendations.

## Review

Materials and methods

Study Design and Search Strategy

This qualitative systematic review was reported in accordance with the Preferred Reporting Items for Systematic Reviews and Meta-Analyses (PRISMA) guidelines. A systematic literature search was conducted in PubMed/MEDLINE to identify studies evaluating EUS-guided drainage of non-pancreatic IAAs using LAMS. No date restrictions were applied. The search was limited to studies published in English and involving adult human patients. Additionally, backward citation searching of the included studies was performed to identify relevant articles not captured in the initial search.

Eligibility Criteria

Studies were considered eligible if they reported original clinical data on EUS-guided drainage of non-pancreatic IAAs using LAMS in adult patients. Acceptable study designs included observational studies, cohort studies, case series, and case reports. Articles focusing exclusively on pancreatic fluid collections were excluded, as were review articles, editorials, conference abstracts without full-text availability, and studies with insufficient or non-separable data specific to non-pancreatic abscesses.

Study Selection and Data Extraction

**Figure 1:**
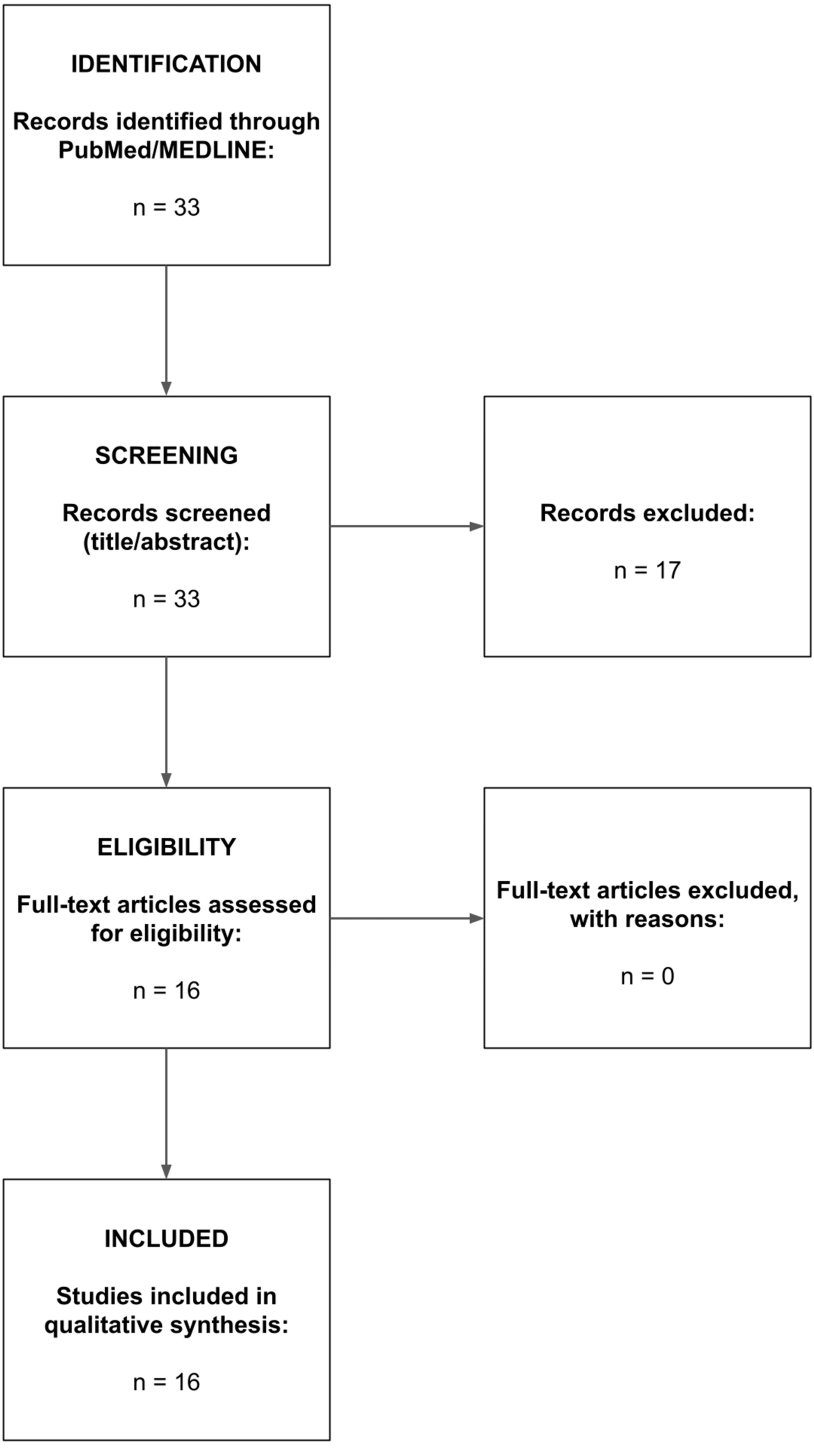
Preferred Reporting Items for Systematic Reviews and Meta-Analyses (PRISMA) flow diagram of study selection. A total of 33 records were identified through PubMed/MEDLINE. After title and abstract screening, 17 records were excluded. Sixteen full-text articles were assessed for eligibility and included in the qualitative synthesis.

Titles and abstracts were screened for relevance, followed by full-text assessment of potentially eligible studies. A total of 33 records were identified through the database search. After title and abstract screening, 17 articles were excluded for not meeting the inclusion criteria. The remaining 16 studies underwent full-text review, all of which met the eligibility criteria and were included in the qualitative synthesis. The study selection process is summarized in the PRISMA flow diagram (Figure 1).

Data were extracted using a standardized approach, including study characteristics, abscess location and etiology, access route, stent characteristics, and reported clinical outcomes.

Outcomes

The primary outcomes were technical success, defined as successful deployment of a LAMS into the abscess cavity, and clinical success, defined as abscess resolution without the need for additional drainage procedures. Secondary outcomes included adverse events, need for reintervention, timing of stent removal, and procedure-related mortality when reported.

Quality Assessment and Data Synthesis

Methodological quality was assessed according to study design using appropriate risk-of-bias assessment tools, including the Joanna Briggs Institute critical appraisal checklists. Due to heterogeneity in study design, abscess characteristics, and reported outcomes, a qualitative synthesis of the available evidence was performed.

Results

Study Selection

A total of 33 records were identified through the systematic literature search in PubMed/MEDLINE. All records were screened at the title and abstract level. After initial screening, 17 studies were excluded for not meeting the predefined inclusion criteria, primarily due to lack of original clinical data, unrelated indications, pancreatic fluid collections, or absence of extractable outcomes specific to non-pancreatic IAAs drainage using LAMS (Appendix). The remaining 16 studies met the eligibility criteria and were included in the qualitative synthesis [8,10-24].

The remaining 16 articles underwent full-text review and met all inclusion criteria. These studies were subsequently included in the qualitative synthesis. No duplicate records were identified during the screening process. The study selection process is summarized in the PRISMA flow diagram (Figure 1).

Study Characteristics

The 16 included studies, published between 2013 and 2024, consisted predominantly of retrospective observational studies, multicenter cohort studies, small case series, and single-patient case reports, with no randomized controlled trials or prospective comparative studies identified [8,10-24]. Sample sizes varied widely, ranging from single-patient case reports to multicenter series including up to 36 patients, reflecting the still limited, although expanding, clinical experience with EUS-guided drainage of non-pancreatic IAAs using LAMS (Table 1) [8,10].

**Table 1:** Characteristics of studies included in the qualitative synthesis (n = 16) LAMS: lumen-apposing metal stent; EC-LAMS: electrocautery-enhanced LAMS; FC-LAMS: fully covered LAMS; EUS: endoscopic ultrasound.

Author (Year)	Study design	n	Abscess type/location	Access route	Main outcomes
Lisotti (2024) [8]	Multicenter retrospective study	36	Pelvic / paracolic abscesses	Transrectal / transcolonic	High technical and clinical success; low adverse event rate
Armellini (2022) [10]	Case report	1	Splenic abscess	Transgastric (EUS-guided)	Successful LAMS drainage with complete clinical resolution
Mudireddy (2017) [11]	Multicenter retrospective study	47	Postoperative intra-abdominal collections	Transgastric / transduodenal / transcolonic (EUS-guided)	High technical and clinical success; acceptable safety profile
Manno (2020) [12]	Case report	1	Intra-abdominal abscess	Transluminal (EUS-guided)	Successful LAMS drainage with complete clinical resolution
Molinaro (2021) [13]	Case report	1	Fungal liver abscess	Transgastric (EUS-guided)	Successful LAMS drainage with complete clinical resolution
Monino (2022) [14]	Case report	1	Diverticulitis-associated pelvic abscess	Transrectal (EUS-guided)	Successful freehand drainage with EC-LAMS; clinical resolution
López Fernández (2019) [15]	Observational case series	3	Postsurgical pelvic abscesses	Transrectal / transcolonic (EUS-guided)	High technical and clinical success with LAMS; acceptable safety profile
Shah (2018) [16]	Case report	1	Multiloculated pelvic abscess (post-appendicitis)	Transrectal / transsigmoid (EUS-guided)	Successful EC-LAMS deployment with immediate purulent drainage and clinical improvement
Machlab (2020) [17]	Case report	1	Subphrenic postoperative collection (post-sleeve gastrectomy)	Transgastric (EUS-guided)	Successful EC-LAMS drainage with rapid clinical improvement and complete resolution
Trindade (2017) [18]	Case report	1	Infected post-surgical intra-abdominal fluid collection	Transluminal (EUS-guided)	Successful LAMS drainage with complete clinical resolution
Attili (2016) [19]	Case report	1	Post-hepatectomy intra-abdominal abscess	Transgastric (EUS-guided, EC-LAMS)	Successful drainage with rapid clinical improvement and abscess resolution
Monino (2021) [20]	Case report	1	Pelvic abscess complicating rectoanal fistula	Transrectal (EUS-guided, EC-LAMS)	Successful drainage with clinical resolution; no major adverse events
Donatelli (2018) [21]	Case report	1	Organized pelvic postoperative collection (post-RYGB)	Transrectal (EUS-guided, FC-LAMS)	Successful LAMS drainage allowing endoscopic necrosectomy; clinical resolution
Alcaide (2013) [22]	Case report	1	Liver abscess	Transgastric (EUS-guided)	Successful LAMS drainage with complete clinical resolution
Hijos (2020) [23]	Case report	1	Intra-abdominal abscess secondary to complicated peptic ulcer	Transgastric (EUS-guided)	Successful LAMS drainage with complete clinical resolution
Simons-Linares (2019) [24]	Case report	1	Subdiaphragmatic abscess (Crohn’s disease)	Transgastric (EUS-guided)	Successful LAMS drainage after failed percutaneous approach; complete resolution

Reported abscess locations included pelvic abscesses, postsurgical intra-abdominal collections, diverticulitis-associated abscesses, hepatic abscesses, splenic abscesses, and subdiaphragmatic abscesses [8,10-24]. Pelvic abscesses, particularly postoperative or diverticulitis-related, represented the most frequently reported indication [8,15,20].

Drainage access routes were tailored according to abscess location and anatomical considerations and included transrectal, transcolonic, transgastric, transduodenal, and transenteric approaches [8,10-24]. Most procedures were performed under EUS guidance, frequently using electrocautery-enhanced LAMS delivery systems [8,10-24].

Technical Success

Technical success, defined as successful deployment of a LAMS within the target abscess cavity, was consistently high across the included studies [8,10-24]. Reported technical success rates ranged from 92% to 100% in larger retrospective and multicenter series [8,10], and reached 100% in nearly all case reports and small case series [11-24].

Clinical Success

Clinical success, defined as complete resolution of the abscess without the need for surgical intervention or additional PD, was achieved in most patients across the included studies [8,10-24]. Reported clinical success rates ranged from approximately 88% to 100% [8,10-24]. In the majority of cases, abscess resolution occurred after a single endoscopic intervention [8,13,16,19].

Stent removal was typically performed within two to six weeks, depending on abscess characteristics, clinical response, and institutional protocols [8,10-24]. Several reports described the use of LAMS to facilitate direct endoscopic necrosectomy in organized or complex collections, particularly in postoperative pelvic abscesses, which contributed to favorable clinical outcomes [15,20,22].

Adverse Events

Adverse events were infrequent and generally mild across the included studies [8,10-24]. Reported complications included transient abdominal or rectal discomfort, minor bleeding, and occasional stent migration [8,10-24]. Serious adverse events were rare, and procedure-related mortality was not reported in any of the included studies [8,10-24]. In larger retrospective and multicenter series, adverse event rates ranged between approximately 3% and 9% [8,10].

Reinterventions

The need for reintervention was uncommon [8,10-24]. When required, reinterventions were predominantly managed endoscopically and included repeat drainage, stent repositioning, or replacement [8,10-24]. In selected cases, additional endoscopic sessions were performed for cavity debridement or necrosectomy [15,20,22]. Limited comparative observations suggested that the use of larger-diameter LAMS may be associated with a reduced number of total endoscopic procedures, although the evidence remains observational and non-comparative [8,10].

Discussion

Principal Findings and Clinical Meaning

In this qualitative systematic review, EUS-guided drainage using LAMS for non-pancreatic IAAs demonstrated consistently high technical success, generally ranging from over 90% to 100%, and high clinical success, typically between approximately 88% and 100% [8,10-24]. Adverse event rates were low, usually below 10%, and most complications were mild and manageable [11-24]. These favorable outcomes may be partially explained by the mechanical advantages of LAMS compared with other devices, such as double-pigtail plastic stents. Specifically, their large inner diameter allows more effective drainage of viscous or infected contents, reducing the risk of stent occlusion, while the biflanged design ensures strong tissue apposition, creating a stable conduit that minimizes peritoneal leakage and stent migration - limitations commonly associated with traditional stents in mobile abdominal compartments.

Collectively, these findings suggest that, in carefully selected patients, LAMS may represent a feasible and effective alternative drainage strategy beyond pancreatic fluid collections. However, their use in this setting remains off-label and is highly dependent on appropriate patient selection, favorable anatomical conditions, and local endoscopic expertise. The current evidence supports LAMS as a complementary tool rather than a replacement for standard percutaneous or surgical approaches [8,10-25].

Why Pelvic Abscesses Dominate the Evidence and Why That Matters

Most of the published experience with EUS-guided LAMS drainage focuses on pelvic abscesses, particularly those related to diverticular disease or postoperative complications. These collections are frequently challenging to access percutaneously and often lie in close apposition to the rectum, sigmoid colon, or distal colon, allowing for a short, stable, and well-visualized transluminal access route under EUS guidance [8,10-24].

This anatomical advantage likely explains both the technical feasibility and the favorable outcomes consistently reported in pelvic collections. Early case reports describing transcolonic or transrectal drainage were followed by larger observational and multicenter experiences demonstrating reproducible results. For example, Fernández-López et al. reported complete abscess resolution without major complications using transcolonic approaches, while Lisotti et al. described high technical and clinical success rates in a multicenter cohort of pelvic and paracolic abscesses [8,10-25].

The concentration of evidence in pelvic abscesses underscores that anatomy, rather than abscess etiology alone, is the primary determinant of success, and it limits the generalizability of current results to other intra-abdominal locations.

Evidence Maturity and Limitations of the Current Literature

Despite encouraging outcomes, the current evidence base remains limited in methodological robustness. Most included studies are retrospective and observational in nature, frequently consisting of small case series or single-patient reports. No randomized controlled trials or prospective comparative studies were identified [8,10-24].

Substantial heterogeneity exists across studies with respect to abscess etiology, anatomical location, access route, technical execution, and outcome definitions. Moreover, patient selection is inherently biased toward cases with a favorable transluminal window and availability of advanced endoscopic expertise, likely contributing to the high reported success rates [8,10-26]

Even the largest multicenter experiences, including those evaluating postsurgical intra-abdominal collections, remain observational and should be interpreted as hypothesis-generating rather than definitive evidence. As such, current data support feasibility and safety in selected scenarios but do not allow firm conclusions regarding superiority over established drainage strategies [8,10-24].

Route Selection and the “Shortest Safe Path” Principle

Across the included studies, favorable outcomes were most consistently reported when drainage followed a short, straight, and well-visualized access route, with clear apposition between the gastrointestinal lumen and the abscess cavity. Transrectal and transcolonic approaches dominated the management of pelvic collections, reflecting both anatomical proximity and procedural stability [8,10-24].

In contrast, transgastric or transduodenal routes were primarily employed for upper abdominal collections, including subdiaphragmatic, hepatic, and splenic abscesses, when close apposition and a safe window could be confirmed. These findings support the principle that route selection should be driven primarily by anatomical feasibility and procedural safety, rather than abscess etiology alone [8,10-26].

The preferred approach should minimize tract length, allow continuous endoscopic ultrasound visualization, avoid intervening vascular structures, and preserve alternative rescue options such as percutaneous or surgical drainage when needed [8,10-24].

Stent Choice and Diameter Considerations

Available evidence suggests that LAMS diameter selection does not substantially affect overall technical or clinical success when appropriate patient selection is applied. Comparative data indicate similar success and safety profiles between 10-mm and 15-mm stents, with some studies suggesting fewer total endoscopic procedures when larger-diameter stents are used [8,10-24].

From a conservative clinical standpoint, stent selection should be individualized, taking into account abscess size, presence of debris or thick purulent material, anticipated need for irrigation or necrosectomy, and the anatomical constraints of the access route [8,10-24].

Safety Profile and Adverse Events

Across the reviewed literature, adverse events related to EUS-guided LAMS drainage of non-pancreatic IAAs were uncommon and generally mild. Reported complications included transient discomfort, minor bleeding, and occasional stent migration. Stent migration, although infrequent, represents a significant clinical concern, particularly in critically ill patients, where a secondary intervention could increase morbidity. In the reviewed literature, cases of migration were predominantly distal (internal) and were managed through endoscopic repositioning or replacement, without the need for emergency surgical salvage. The biflanged, saddle-shaped design of LAMS is specifically engineered to provide an anchoring effect, which likely contributes to the low migration rates observed compared to straight metal or plastic stents. However, to further minimize this risk, meticulous pre-procedural planning is essential to ensure adequate wall-to-wall apposition, as large distances between the lumen and the collection are the primary driver of displacement. Procedure-related mortality was not reported [8,10-24].

Nevertheless, serious LAMS-related complications are well recognized in the broader endoscopic literature, including bleeding, buried stent syndrome, stent occlusion, and perforation. These risks reinforce the importance of a cautious and selective approach, with procedures performed in centers capable of close post-procedural monitoring and prompt endoscopic, radiologic, or surgical rescue when required [5-7].

Proposed Conservative, Anatomy-Driven Practical Approach

**Figure 2:**
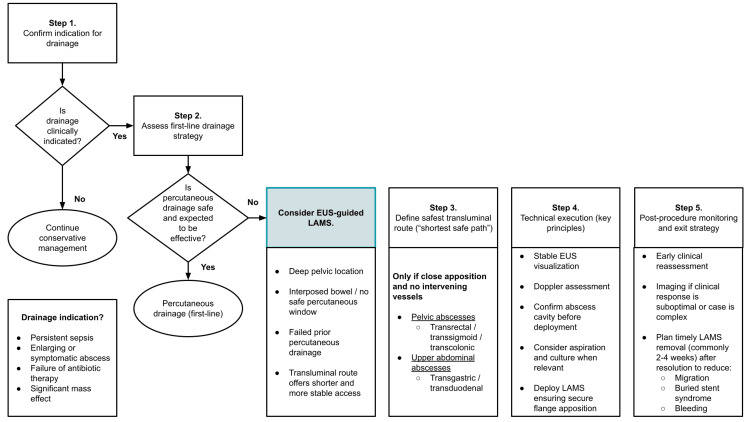
Proposed conservative, anatomy-driven practical approach for EUS-guided lumen-apposing metal stent drainage of non-pancreatic intra-abdominal abscesses. EUS, endoscopic ultrasound; LAMS, lumen-apposing metal stent. This schematic illustrates a pragmatic, stepwise decision-making framework for the use of EUS guided LAMS in selected patients with non-pancreatic intra-abdominal abscesses. This algorithm is intended to assist clinical judgment and multidisciplinary decision-making and does not represent a formal guideline.

Based on the findings of this qualitative systematic review and acknowledging the limitations of the available evidence, a conservative, anatomy-driven, practical approach for the use of EUS-guided LAMS in selected patients with non-pancreatic IAAs is proposed. This approach is intended to assist clinical decision-making rather than to serve as a formal guideline (Figure 2) [8,10-24].

First, drainage should be considered only when a clear clinical indication is present. These indications include inadequate response to antibiotic therapy (manifested as persistent sepsis), enlarging collections, or symptomatic abscesses causing significant mass effect. Whenever feasible, decision-making should involve a multidisciplinary discussion including gastroenterology, interventional radiology, and surgery to ensure that all available therapeutic options have been appropriately considered [1-3].

Second, standard image-guided PD should remain the first-line approach when it is feasible and expected to be effective. EUS-guided LAMS placement may be considered in selected scenarios, particularly when the percutaneous route is not feasible due to deep pelvic location, interposed bowel, or lack of a safe access window, when prior PD has failed, or when a transluminal approach offers a shorter and more stable access route in experienced centers [8,10-24].

Third, a careful definition of the safest transluminal access route is essential and should follow the principle of selecting the shortest and safest path between the gastrointestinal lumen and the abscess cavity. For pelvic collections, transrectal, transsigmoid, or transcolonic routes are most commonly supported by the available evidence. In contrast, upper abdominal collections, including subdiaphragmatic or hepatic infected collections, may be approached via transgastric or transduodenal routes when close apposition is confirmed and intervening vascular structures can be safely avoided [8,10-24].

Fourth, technical execution should emphasize stable EUS visualization, meticulous Doppler assessment to avoid intervening vessels, and confirmation of the abscess cavity prior to stent deployment. Aspiration and microbiological sampling may be performed when clinically relevant. LAMS should be deployed with secure flange positioning. Adjunctive measures such as lavage or placement of a coaxial plastic stent should be reserved for selected cases, recognizing that most supporting data for these strategies originate from pancreatic fluid collection literature and broader LAMS safety and technical guidance [5-7].

Finally, close post-procedural monitoring is essential. Early clinical reassessment for fever, pain, or laboratory abnormalities should be performed, and cross-sectional imaging should be obtained when clinical response is suboptimal or in complex cases. A clearly defined exit strategy, including timely stent removal after abscess resolution, should be planned to reduce the risk of delayed adverse events such as stent migration, buried stent syndrome, or bleeding [5-7].

## Conclusions

EUS-guided drainage using LAMS appears to be a feasible and effective therapeutic option for carefully selected patients with non-pancreatic IAAs, particularly in anatomically challenging scenarios where PD is unfeasible or has failed. However, it is essential to note that these high success rates are derived from highly selected populations treated in specialized, high-volume centers, which may limit the generalizability of these findings to broader clinical settings.

While the available literature reports favorable outcomes and a low incidence of adverse events, the evidence remains limited by its retrospective and observational nature. Consequently, the anatomy-driven approach proposed in this review should be regarded as an expert-informed framework intended to support clinical decision-making, rather than standardized, evidence-based guidance. Further prospective and comparative studies are mandatory to define the definitive role of LAMS and to establish formal clinical guidelines for non-pancreatic IAA management.

## Appendices

**Table 2:** Studies excluded after full-text review and reasons for exclusion LAMS: lumen-apposing metal stent; EUS: endoscopic ultrasound.

PMID	Author (Year)	Reason for exclusion
29519336	Mussetto (2018)	Review article without original clinical data.
37934190	International Delphi Consensus	Consensus guideline without original clinical data.
38145052	Huang (2023)	Unrelated topic; not involving lumen-apposing metal stents or intra-abdominal abscess drainage.
31830600	Tafaj (2020)	Unrelated topic; not involving lumen-apposing metal stents or intra-abdominal abscess drainage.
34297256	Spota (2021)	Mixed surgical complication population without extractable data specific to non-pancreatic intra-abdominal abscess drainage using LAMS.
36259175	Yamamoto (2023)	Pancreatic fluid collection; peripancreatic indication outside the scope of non-pancreatic intra-abdominal abscess drainage.
18199794	Valcheva (2008)	Unrelated topic; microbiological epidemiology study not involving intra-abdominal abscesses or LAMS.
34565279	Guingand (2021)	Mixed endoscopic interventions with limited LAMS use and no extractable LAMS-specific outcomes.
28196390	Poincloux (2017)	Mixed endoscopic interventions with limited LAMS use and no extractable LAMS-specific outcomes.
32023636	Troncone (2020)	Pancreatic fluid collection and management of stent-related complications; outside the scope of non-pancreatic intra-abdominal abscess drainage.
30238760	Ortiz Moyano (2018)	Pancreatic fluid collection indication; outside the scope of non-pancreatic intra-abdominal abscess drainage using LAMS.
33931322	Donatelli (2021)	Mixed postoperative adverse event population with limited collection cases and no extractable LAMS-specific outcomes for non-pancreatic intra-abdominal abscess drainage.
32267555	Matsubara (2020)	Different indication and intervention: EUS-guided gallbladder drainage for acute cholecystitis without LAMS use.
39788214	Ichkhanian (2025)	Mixed pancreatic and non-pancreatic intra-abdominal abscess population without extractable outcomes specific to non-pancreatic abscess drainage.
17921395	Clarke (2007)	Unrelated topic; metabolic study not involving intra-abdominal abscesses or LAMS.
24929485	Consiglieri (2014)	Extra-abdominal (mediastinal) abscess; outside the scope of intra-abdominal abscess drainage.
33428037	Pagano (2021)	Unrelated topic; mediastinal foreign body removal without intra-abdominal abscess drainage or LAMS use.
